# On Eccentricity-Based Topological Indices Study of a Class of Porphyrin-Cored Dendrimers

**DOI:** 10.3390/biom8030071

**Published:** 2018-08-07

**Authors:** Wei Gao, Zahid Iqbal, Muhammad Ishaq, Rabia Sarfraz, Muhammad Aamir, Adnan Aslam

**Affiliations:** 1 School of Information Science and Technology, Yunnan Normal University, Kunming 650500, China; gaowei@ynnu.edu.cn; 2 School of Natural Sciences, National University of Sciences and Technology, Sector H-12, Islamabad 44000, Pakistan; 786zahidwarraich@gmail.com (Z.I.); ishaq_maths@yahoo.com (M.I.); sarfaraz_rabia@yahoo.com (R.S.); 3 Faculty of Physical and Numerical Sciences, Abdul Wali Khan University Mardan, Mardan, Pakistan; aamirkhan@awkum.edu.pk; 4 Department of Mathematical Sciences, Faculty of Science, Universiti Teknologi Malaysia, Johor Bahru 81310, Malaysia; 5 Department of Natural Sciences and Humanities, University of Engineering and Technology, Lahore 54000, Pakistan

**Keywords:** porphyrin-cored dendrimers, eccentric connectivity index, total eccentric connectivity index, augmented eccentric connectivity index

## Abstract

It is revealed from the previous studies that there is a strong relation between the chemical characteristic of a chemical compound and its molecular structure. Topological indices defined on the molecular structure of biomolecules can help to gain a better understanding of their physical features and biological activities. Eccentricity connectivity indices are distance-based molecular structure descriptors that have been used for the mathematical modeling of biological activities of diverse nature. As the porphyrin has photofunctional properties, such as a large absorption cross-section, fluorescence emission, and photosensitizing properties, due to these properties, porphyrin dendrimers can be used as photofunctional nanodevices. In this paper, we compute the exact formulae of different versions of eccentric connectivity index and their corresponding polynomials for a class of porphyrin-cored dendrimers. The results obtained can be used in computer-aided molecular design methods applied to pharmaceutical engineering.

## 1. Introduction

In this era of rapid technological development, a large number of new nanomaterials, crystalline materials, and drugs emerge every year. To determine the chemical properties of such a large number of new compounds and new drugs requires a large amount of chemical experiments, thereby greatly increasing the workload of the chemical and pharmaceutical researchers. In this regard, computing different types of topological indices has provided the insights into medicinal behaviour of several compounds and drugs [1,2]. The computation method of topological indices has proven its worth by yielding medical information of drugs with less use of chemical-related equipment [3,4].

In chemical graph theory, various graphical invariants are used for establishing correlations of chemical structures with various physical properties, chemical reactivity, or biological activity [5,6]. These graphical invariants are called topological indices of molecular graphs in this field. There is a large family of distance-based topological indices of molecular graphs in chemical graph theory. In chemistry, biochemistry and nanotechnology, distance-based topological indices of a graph are found to be useful in isomer discrimination, structure–property relationships and structure–activity relationships [7,8].

Dendrimers are synthetic polymers, of diverse initiator core, branches and the peripheral groups have been extensively synthesized in the last two decades [9]. Numerous kinds of experiments have proven that these polymers with well-defined structures and topological architectures exhibited an array of applications in medicine [10]. In these times, dendrimers are inviting the interest of a great number of researchers because of their remarkable physical and chemical properties and the wide range of promising applications in different fields [11,12,13]. Until now, the study of the eccentricity based topological indices for special chemical and nanostructures has been largely limited. Thus, we have been attracted to studying the mathematical properties of the eccentricity based topological indices and their polynomial versions of a class of dendrimers.

Now, we introduce some notations and definitions, which will be needed throughout this monograph. In a theoretical chemistry setting, chemical compounds are expressed as graphs. Let G=(V(G),E(G)) be a molecular graph, where V(G), and E(G) appear for the vertex and edge set, respectively. Vertices of *G* correlate with the atoms, and edges correspond to the chemical bonds between atoms. In a graph *G*, if two vertices *s* and *t* are end vertices of an edge e∈E(G), then they are called adjacent, and we write this edge as e=st or e=ts. For a vertex *t*, the set of neighbor vertices is denoted by $N_{t}$ and is defined as $N_{t}=\left\{s\in V\left(G\right):st\in E\left(G\right)\right\}$. The degree of a vertex t∈V(G) is represented by $d_{t}$ and is expressed as the number of edges connecting it. Let $S_{t}$ denote the sum of the degrees of neighboring vertices of vertex *t*, which is $S_{t}=\sum_{s\in N_{t}}d_{s}$. A $\left(t_{1},t_{n}\right)$-path on *n* vertices is defined as a graph with vertex set $\left\{t_{1},\cdots ,t_{n}\right\}$ and edge set $\left\{t_{i}t_{i+1}:1\leq i\leq n-1\right\}$. The distance between two vertices s,t∈V(G) is represented by d(s,t) and defined as the length of the shortest (s,t)-path in *G*. For a given vertex t∈V(G), the eccentricity ε(t) is defined as the maximum distance between *t* and any other vertex in *G*.

The history of topological indices goes back to 1947, when Harold Wiener put forward the study of first distance-based topological index, known as Wiener index [14]. Sharma, Goswami, and Madan [15] initiated the study of another distance-based topological index, termed as the eccentric connectivity index, of the graph *G*, described as

(1) $$\xi \left(G\right)=\sum_{u\in V(G)}^{}\varepsilon \left(u\right)d_{u},$$

which has been employed usefully for the buildup of various mathematical models for the prediction of biological activities of distinct nature. Some applications and mathematical properties of this index have been discussed by several authors [16,17,18,19]. For a graph *G*, Ashrafi and Jalali extended the study of the eccentric connectivity index by introducing the eccentric connectivity polynomial in [20]:

(2) $$ECP\left(G,y\right)=\sum_{u\in V(G)}d_{u}y^{\varepsilon (u)}.$$

If we consider only the eccentricity of the vertices, then we obtain the total eccentricity index of the graph *G*, which is expressed as follows:

(3) $$\varsigma \left(G\right)=\sum_{t\in V(G)}\varepsilon \left(t\right).$$

For a graph *G*, the polynomial version of the total eccentricity index is described by Ashrafi et al. [20]:

(4) $$TECP\left(G,x\right)=\sum_{t\in V(G)}x^{\varepsilon (t)}.$$

Ghorbani and Hosseinzadeh in [21] presented the first Zagreb index of a graph *G* in terms of eccentricity by replacing the vertex degrees with the vertex eccentricities in the following way:

(5) $$M_{1}\left(G\right)=\sum_{t\in V(G)}{\left(\varepsilon \left(t\right)\right)}^{2}.$$

Gupta, Singh and Madan [22] introduced the augmented eccentric connectivity index of a graph *G*, as a generalization of the eccentric connectivity index as follows:

(6) $${}^{A}\varepsilon \left(G\right)=\sum_{t\in V(G)}\frac{M(t)}{\varepsilon (t)},$$

where M(t) represents the product of degrees of all neighbors of vertex *t*. Various properties of this index have been examined in previous articles [23,24,25]. For a graph *G*, the modified versions of eccentric connectivity index and polynomial are defined as follows:

(7) $$\Lambda \left(G\right)=\sum_{t\in V(G)}S_{t}\varepsilon \left(t\right),$$

(8) $$MECP\left(G,x\right)=\sum_{t\in V(G)}S_{t}x^{\varepsilon (t)}.$$

**Figure 1:**
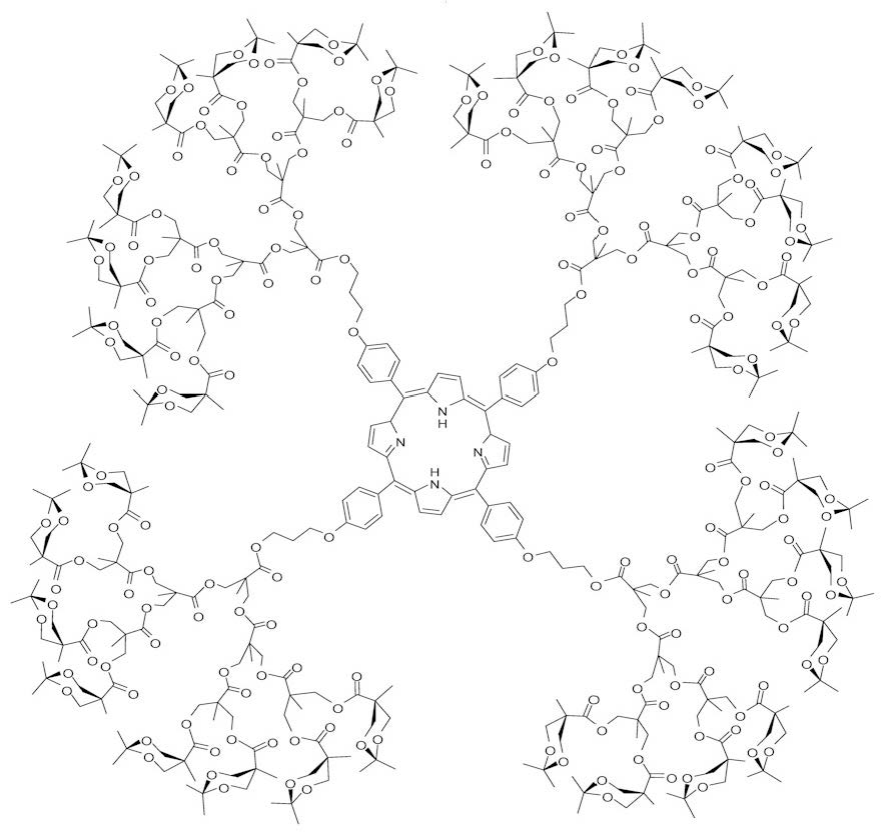
Porphyrin-cored 2,2-bis (methylol) propionic acid dendrimers for n=4.

Several mathematical and chemical properties of these modified versions have been studied [4,20]. For detailed results on the topological indices of graphs, the readers may refer to the previous reports [26,27,28,29,30,31,32,33]. As the porphyrin has unique photofunctional properties, such as a fluorescence emission, large absorption cross-section, and photosensitizing properties; so porphyrin dendrimers can be used as photofunctional nano-devices. The high solubility of porphyrin dendrimers permits their use in photodynamic therapy, a promising technology for fewer invasive cancer treatments [34]. In this paper, we will study the different topological indices and polynomials of the molecular graph of porphyrin-cored 2,2-bis (methylol) propionic acid dendrimers. Figure 1 depicts the molecular graph of the porphyrin-cored 2,2-bis (methylol) propionic acid dendrimer with the fourth growth stage. The synthesis and characterization of this dendrimer are described by Vestberg et al. (see [35]).

## 2. The Eccentricity-Based Indices and Polynomials for the Molecular Graph

**Figure 2:**
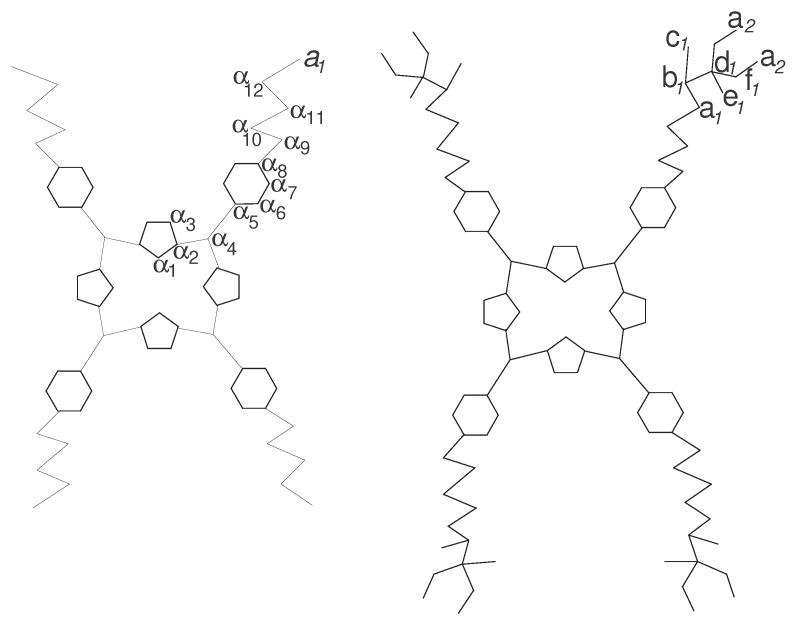
Core of the dendrimer D(n); first generation of the dendrimer D(n).

A hydrogen-depleted molecular graph is the molecular graph with hydrogen vertices deleted. The characterization of a molecule by an associated graph leads to a large number of powerful and useful discriminators called topological indices. In chemical graph theory, usually a topological index is a numerical parameter mathematically derived from the hydrogen-suppressed molecular graph [36,37,38]. Although it is customary to use hydrogen-suppressed molecular graphs, there exist graph descriptors and topological indices that are computed from the whole, hydrogen-containing molecular graph [39]. We denote the hydrogen-suppressed molecular graph of this dendrimer with D(n), where the generation stage of D(n) is represented by *n* (see Figure 1). The graph of the dendrimer D(n) consists of a central core with 68 vertices and four branches with each branch having $2^{3}\left(1+2+2^{2}+\cdots +2^{n-1}\right)=2^{n+3}-8$ vertices. Hence, a simple calculation shows that the order of D(n) is $4(9+2^{n+3})$. In addition, each branch consist of $2^{n+3}-8$ edges. This gives the total number of edges in D(n) are $4(11+2^{n+3})$. To work out the eccentricity-based indices and polynomials of D(n), it is sufficient to determine the desired data for the sets of representatives. We partition the molecular graph D(n) into two parts, one of them is core *C* and other is subgraph Z(n) of D(n) having four similar branches with vertex set V(D(n))-V(C). The molecular graphs of cores and the first generation of dendrimers are shown in Figure 2. The sets of representatives from a set of vertices of the core and subgraph of D(n), with their degrees, $S_{t}$, M(t) and eccentricity for each *t* are given in Table 1 and Table 2, respectively. For the core, these representatives are labelled by $\alpha_{l}$, where 1≤l≤12 as shown in Figure 2. For Z(n), where n≥1, these representatives are labelled by $a_{i}$, $b_{i}$, $c_{i}$, $d_{i}$, $e_{i}$, $f_{i}$, $a_{n+1}$, where 1≤i≤n as shown in Figure 2. For our convenience, throughout this paper, we use γ=4n+4i.

By using Table 1 and Table 2, we calculate the different eccentricity-based indices and their corresponding polynomials. In the following theorem, we determine the eccentric connectivity index of D(n).

**Theorem** **1.** *The eccentric connectivity index for the molecular graph D(n) is given by*

$$\xi \left(D\left(n\right)\right)=16\left(2^{n+5}\times n+22n+2^{n}\times 81+101\right).$$

**Proof.**  By using the values of Table 1 and Table 2 in the definition of eccentricity connectivity index given by Equation (1), the eccentric connectivity index of D(n) can be computed as follows:

$$\begin{array}{c} \;\xi \left(D\left(n\right)\right)=\xi \left(C\right)+\xi \left(Z\left(n\right)\right)=\sum_{t\in V(C)}\varepsilon \left(t\right)d_{t}+\sum_{t\in V(Z(n))}\varepsilon \left(t\right)d_{t}\\ =(2\times 4)(4n+15)+(3\times 8)(4n+16)+(2\times 8)(4n+16)+(3\times 4)(4n+17)\\ +(3\times 4)(4n+18)+(2\times 8)(4n+19)+(2\times 8)(4n+20)+(3\times 4)(4n+21)\\ +(2\times 4)(4n+22)+(2\times 4)(4n+23)+(2\times 4)(4n+24)+(2\times 4)(4n+25)\\ +\left(1\times 2^{n+2}\right)\left(8n+26\right)+\sum_{i=1}^{n}(\left(2\times 2^{i+1}\right)\left(\gamma +22\right)+\left(3\times 2^{i+1}\right)\left(\gamma +23\right)\\ +\left(1\times 2^{i+1}\right)\left(\gamma +24\right)+\left(4\times 2^{i+1}\right)\left(\gamma +24\right)+\left(1\times 2^{i+1}\right)\left(\gamma +25\right)+\left(2\times 2^{i+2}\right)\left(\gamma +25\right)). \end{array}$$

After some calculations, we get

$$\xi \left(D\left(n\right)\right)=16\left(2^{n+5}\times n+22n+2^{n}\times 81+101\right),$$

which proves the theorem. □

In the similar way, by putting the values of Table 1 and Table 2 in Equation (3), we have the following result.

**Corollary** **1.** *The total eccentric connectivity index of D(n) is given by*

$$\varsigma \left(D\left(n\right)\right)=4\left(2^{n+6}\times n+2^{n+1}\times 83+36n+167\right).$$

In the upcoming theorem, the absolute formula for the eccentric connectivity polynomial for D(n) has been obtained.

**Theorem** **2.** *The eccentric connectivity polynomial for D(n) is given by*

$$\begin{array}{c} \begin{array}{cc} ECP(D(n),x) & =4x^{4n+15}(2x^{10}+2x^{9}+2x^{8}+2x^{7}+3x^{6}+4x^{5}+4x^{4}+3x^{3}+3x^{2}\\ & +10x+2)+2^{n+2}x^{2(4n+13)}+\frac{4\left(5x^{3}+5x^{2}+3x+2\right)\times x^{4n+26}\left(2^{n}x^{4n}-1\right)}{2x^{4}-1}. \end{array} \end{array}$$

**Proof.**  By using the values of Table 1 and Table 2 in Equation (2), we have

$$\begin{array}{c} \begin{array}{cc} ECP(D(n),x) & =ECP\left(C,x\right)+ECP\left(Z\left(n\right),x\right)=\sum_{t\in V(C)}d_{t}x^{\varepsilon (t)}+\sum_{t\in V(Z(n))}d_{t}x^{\varepsilon (t)}\\ & =\left(2\times 4\right)x^{4n+15}+\left(8\times 3\right)x^{4n+16}+\left(8\times 2\right)x^{4n+16}+\left(4\times 3\right)x^{4n+17}\\ & +\left(4\times 3\right)x^{4n+18}+\left(8\times 2\right)x^{4n+19}+\left(8\times 2\right)x^{4n+20}+\left(4\times 3\right)x^{4n+21}\\ & +\left(4\times 2\right)x^{4n+22}+\left(4\times 2\right)x^{4n+23}+\left(4\times 2\right)x^{4n+24}+\left(4\times 2\right)x^{4n+25}\\ & +\left(1\times 2^{n+2}\right)x^{8n+26}+\sum_{i=1}^{n}(\left(2\times 2^{i+1}\right)x^{\gamma +22}+\left(3\times 2^{i+1}\right)x^{\gamma +23}\\ & +\left(1\times 2^{i+1}\right)x^{\gamma +24}+\left(4\times 2^{i+1}\right)x^{\gamma +24}+\left(1\times 2^{i+1}\right)x^{\gamma +25}+\left(2\times 2^{i+2}\right)x^{\gamma +25}). \end{array} \end{array}$$

After several calculation steps, we obtain the desired result. □

The total eccentric connectivity polynomial of D(n) can be calculated by using the values of Table 1 and Table 2 in Equation (4). We have the following result.

**Corollary** **2.** *The total eccentric connectivity polynomial of D(n) is given by*

$$\begin{array}{c} \begin{array}{cc} TECP(D(n),x & )=4x^{4n+15}\left(x^{10}+x^{9}+x^{8}+x^{7}+x^{6}+2x^{5}+2x^{4}+x^{3}+x^{2}+4x+1\right)\\ & +2^{n+2}x^{2(4n+13)}+\frac{4\left(3x^{3}+2x^{2}+x+1\right)x^{4n+26}\left(2^{n}x^{4n}-1\right)}{2x^{4}-1}. \end{array} \end{array}$$

Now, we compute the precise value of first Zagreb eccentricity index.

**Theorem** **3.** *The first Zagreb eccentricity index for the molecular graph D(n) is given by*

$$M_{1}\left(D\left(n\right)\right)=4\left(2^{n+9}\times n^{2}+2^{n+5}\times 83n+2^{n+2}\times 927+144n^{2}+1336n+3007\right).$$

**Proof.**  By using the values of Table 1 and Table 2 in Equation 5, we compute the first Zagreb eccentricity index of D(n) as follows:

$$\begin{array}{c} \begin{array}{cc} M_{1}\left(D\left(n\right)\right) & =M_{1}\left(C\right)+M_{1}\left(Z\left(n\right)\right)=\sum_{v\in V(C)}{\left[\varepsilon \left(v\right)\right]}^{2}+\sum_{v\in V(Z(n))}{\left[\varepsilon \left(v\right)\right]}^{2}\\ & =4{\left(4n+15\right)}^{2}+8{\left(4n+16\right)}^{2}+8{\left(4n+16\right)}^{2}+4{\left(4n+17\right)}^{2}+4{\left(4n+18\right)}^{2}\\ & +8{\left(4n+19\right)}^{2}+8{\left(4n+20\right)}^{2}+4{\left(4n+21\right)}^{2}+4{\left(4n+22\right)}^{2}+4{\left(4n+23\right)}^{2}\\ & +4{\left(4n+24\right)}^{2}+4{\left(4n+25\right)}^{2}+2^{n+2}{\left(8n+26\right)}^{2}+\sum_{i=1}^{n}(2^{i+1}{\left(\gamma +22\right)}^{2}\\ & +2^{i+1}{\left(\gamma +23\right)}^{2}+2^{i+1}{\left(\gamma +24\right)}^{2}+2^{i+1}{\left(\gamma +24\right)}^{2}+2^{i+1}{\left(\gamma +25\right)}^{2}+2^{i+2}{\left(\gamma +25\right)}^{2}). \end{array} \end{array}$$

After some calculations, we have

$$M_{1}\left(D\left(n\right)\right)=4\left(2^{n+9}\times n^{2}+2^{n+5}\times 83n+2^{n+2}\times 927+144n^{2}+1336n+3007\right),$$

which proves our theorem. □

**Theorem** **4.** *The augmented eccentric connectivity index for the molecular graph D(n) is given by*

$$\begin{array}{c} \begin{array}{cc} {}^{A}\varepsilon \left(D\left(n\right)\right) & =\frac{36}{4n+15}+\frac{144}{4n+16}+\frac{108}{4n+17}+\frac{48}{4n+18}+\frac{48}{4n+19}+\frac{48}{4n+20}\\ & +\frac{32}{4n+21}+\frac{24}{4n+22}+\frac{16}{4n+23}+\frac{16}{4n+24}+\frac{8}{4n+25}+\frac{2^{n+3}}{8n+26}\\ & +\left(\frac{24}{4n+26}+\cdots +\frac{3\times 2^{n+2}}{8n+22}\right)+\left(\frac{32}{4n+27}+\cdots +\frac{2^{n+4}}{8n+23}\right)\\ & +\left(\frac{12}{4n+28}+\cdots +\frac{3\times 2^{n+1}}{8n+24}\right)+\left(\frac{48}{4n+28}+\cdots +\frac{3\times 2^{n+3}}{8n+24}\right)\\ & +\left(\frac{16}{4n+29}+\cdots +\frac{2^{n+3}}{8n+25}\right)+\left(\frac{32}{4n+29}+\cdots +\frac{2^{n+4}}{8n+25}\right). \end{array} \end{array}$$

**Proof.**  According to the values given in Table 1 and Table 2 and Equation (6), we work out the augmented eccentric connectivity index of D(n) in the following way:

$$\begin{array}{c} \begin{array}{cc} {}^{A}\varepsilon \left(D\left(n\right)\right) & {=}^{A}\varepsilon \left(C\right){+}^{A}\varepsilon \left(Z\left(n\right)\right)=\sum_{t\in V(C)}\frac{M(t)}{\varepsilon (t)}+\sum_{t\in V(Z(n))}\frac{M(t)}{\varepsilon (t)}\\ & =\frac{9\times 4}{4n+15}+\frac{12\times 8}{4n+16}+\frac{6\times 8}{4n+16}+\frac{27\times 4}{4n+17}+\frac{12\times 4}{4n+18}+\frac{6\times 8}{4n+19}\\ & +\frac{6\times 8}{4n+20}+\frac{4\times 8}{4n+21}+\frac{6\times 4}{4n+22}+\frac{4\times 4}{4n+23}+\frac{4\times 4}{4n+24}+\frac{2\times 4}{4n+25}+\frac{2\times 2^{n+2}}{8n+26}\\ & +\sum_{i=1}^{n}\left(\frac{6\times 2^{i+1}}{\gamma +22}+\frac{8\times 2^{i+1}}{\gamma +23}+\frac{3\times 2^{i+1}}{\gamma +24}+\frac{12\times 2^{i+1}}{\gamma +24}+\frac{4\times 2^{i+1}}{\gamma +25}+\frac{4\times 2^{i+2}}{\gamma +25}\right). \end{array} \end{array}$$

After merging up the similar items in the above equation, we obtain the required result. □

Now, we determine the accurate value of the modified eccentric connectivity index.

**Theorem** **5.** *For D(n), the modified eccentric connectivity index is given by*

$$\Lambda \left(D\left(n\right)\right)=4\left(2^{n+3}\times 39n+204n+2^{n}\times 789+905\right).$$

**Proof.**  By inserting the values of Table 1 and Table 2 in Equation (7), we determine the modified eccentric connectivity index of D(n) as follows:

$$\begin{array}{c} \begin{array}{cc} \Lambda (D(n)) & =\Lambda \left(C\right)+\Lambda \left(Z\left(n\right)\right)=\sum_{t\in V(C)}S_{t}\varepsilon \left(t\right)+\sum_{t\in V(Z(n))}S_{t}\varepsilon \left(t\right)\\ & =(6\times 4)(4n+15)+(7\times 8)(4n+16)+(5\times 8)(4n+16) \end{array} \end{array}$$

$$\begin{array}{c} \begin{array}{c} +(9\times 4)(4n+17)+(7\times 4)(4n+18)+(5\times 8)(4n+19)+(5\times 8)(4n+20)\\ +(6\times 4)(4n+21)+(5\times 4)(4n+22)+(4\times 4)(4n+23)+(4\times 4)(4n+24)\\ +\left(3\times 4\right)\left(4n+25\right)+\left(2\times 2^{n+2}\right)\left(8n+26\right)+\sum_{i=1}^{n}(\left(5\times 2^{i+1}\right)\left(\gamma +22\right)+\left(7\times 2^{i+1}\right)\left(\gamma +23\right)\\ +\left(3\times 2^{i+1}\right)\left(\gamma +24\right)+\left(8\times 2^{i+1}\right)\left(\gamma +24\right)+\left(4\times 2^{i+1}\right)\left(\gamma +25\right)+\left(5\times 2^{i+2}\right)\left(\gamma +25\right)). \end{array} \end{array}$$

Clearly, the above equation is equal to

$$\Lambda \left(D\left(n\right)\right)=4\left(2^{n+3}\times 39n+204n+2^{n}\times 789+905\right),$$

which implies the desired result. □

In the following theorem, we compute the closed formula for the modified eccentric connectivity polynomial.

**Theorem** **6.** *The modified eccentric connectivity polynomial of D(n) is given by*

$$\begin{array}{c} \Lambda \left(D\left(n\right)\right)=4x^{4n+15}(3x^{10}+4x^{9}+4x^{8}+5x^{7}+6x^{6}+10x^{5}+10x^{4}+7x^{3}\\ +9x^{2}+24x+6)+2^{n+3}x^{2(4n+13)}+\frac{4\left(14x^{3}+11x^{2}+7x+5\right)x^{4n+26}\left(2^{n}x^{4n}-1\right)}{2x^{4}-1}. \end{array}$$

**Proof.**  Followed by the values depicted in Table 1 and Table 2, and the expression of the modified eccentric connectivity polynomial in Equation (8), the value of modified eccentric connectivity polynomial of D(n) can be written as follows:

$$\begin{array}{cc} MECP(D(n),x) & =MECP\left(C,x\right)+MECP\left(Z\left(n\right),x\right)=\sum_{t\in V(C)}S_{t}x^{\varepsilon (t)}+\sum_{t\in V(Z(n))}S_{t}x^{\varepsilon (t)}\\ & =\left(6\times 4\right)x^{4n+15}+\left(8\times 7\right)x^{4n+16}+\left(8\times 5\right)x^{4n+16}+\left(4\times 9\right)x^{4n+17}\\ & +\left(4\times 7\right)x^{4n+18}+\left(8\times 5\right)x^{4n+19}+\left(8\times 5\right)x^{4n+20}+\left(4\times 6\right)x^{4n+21}\\ & +\left(4\times 5\right)x^{4n+22}+\left(4\times 4\right)x^{4n+23}+\left(4\times 4\right)x^{4n+24}+\left(4\times 3\right)x^{4n+25}\\ & +\left(2\times 2^{n+2}\right)x^{8n+26}+\sum_{i=1}^{n}(\left(5\times 2^{i+1}\right)x^{\gamma +22}+\left(7\times 2^{i+1}\right)x^{\gamma +23}\\ & +\left(3\times 2^{i+1}\right)x^{\gamma +24}+\left(8\times 2^{i+1}\right)x^{\gamma +24}+\left(4\times 2^{i+1}\right)x^{\gamma +25}+\left(5\times 2^{i+2}\right)x^{\gamma +25}). \end{array}$$

By means of simple calculations, we obtain the required result. □

## 3. Conclusions

With the remarkable growth in the field of computer technology and accompanied by the introduction of the utilization of it in pharmacology, chemistry, and biology, a set of methodologies like quantitative structure–activity relationship and quantitative structure-property relationships have been developed. These methodologies have been widely applied in medicinal chemistry for the interpretation of many chemical and biological processes. In this paper, by molecular structure analysis, eccentricity calculating and mathematical derivation, we have computed the precise values of different versions of eccentric connectivity indices and their corresponding polynomials for a class of porphyrin-cored dendrimers. The theoretical formulations obtained in our work illustrate the promising prospects of their application for the pharmacy and chemical engineering.

## Figures and Tables

**Table 1:** The set of representatives of the core with their degrees, $S_{t}$, M(t), eccentricities and frequencies.

Representative	Degree	$S_{t}$	M(t)	Eccentricity	Frequency
$\alpha_{1}$	2	6	9	4n+15	4
$\alpha_{2}$	3	7	12	4n+16	8
$\alpha_{3}$	2	5	6	4n+16	8
$\alpha_{4}$	3	9	27	4n+17	4
$\alpha_{5}$	3	7	12	4n+18	4
$\alpha_{6}$	2	5	6	4n+19	8
$\alpha_{7}$	2	5	6	4n+20	8
$\alpha_{8}$	3	6	8	4n+21	4
$\alpha_{9}$	2	5	6	4n+22	4
$\alpha_{10}$	2	4	4	4n+23	4
$\alpha_{11}$	2	4	4	4n+24	4
$\alpha_{12}$	2	3	2	4n+25	4

**Table 2:** The set of representatives of the subgraph Z(n) of D(n) with degrees, $S_{t}$, M(t), eccentricities and frequencies.

Representative	Degree	$S_{t}$	M(t)	Eccentricity	Frequency
$a_{n+1}$	1	2	2	8n+26	$2^{n+2}$
$a_{i}$	2	5	6	γ+22	$2^{i+1}$
$b_{i}$	3	7	8	γ+23	$2^{i+1}$
$c_{i}$	1	3	3	γ+24	$2^{i+1}$
$d_{i}$	4	8	12	γ+24	$2^{i+1}$
$e_{i}$	1	4	4	γ+25	$2^{i+1}$
$f_{i}$	2	5	4	γ+25	$2^{i+2}$
